# Detection of AZF microdeletions and analysis of reproductive hormonal profiles in Hainan men undergoing assisted reproductive technology

**DOI:** 10.1186/s12894-024-01503-x

**Published:** 2024-06-12

**Authors:** Qina He, Yongle Zhang, Mengyi Song, Yao Zhou, Dan Lin, Yanlin Ma, Fei Sun, Qi Li

**Affiliations:** 1 Hainan Provincial Key Laboratory for Human Reproductive Medicine and Genetic Research, Hainan Provincial Clinical Research Center for Thalassemia, the Key Laboratory of Tropical Translational Medicine of Ministry of Education, Department of Reproductive Medicine, the First Affiliated Hospital of Hainan Medical University, Hainan Medical University, Haikou, Hainan 571101 China; 2 Haikou Key Laboratory for Preservation of Human Genetic Resource, the First Affiliated Hospital of Hainan Medical University, Haikou, Hainan 571101 China; 3 Department of Obstetrics and Gynecology, Reproductive Medicine, Nanfang Hospital, Southern Medical University, Guangdong, 510515 China; 4 Hainan Modern Women and Children’s Hospital, Reproductive Medicine, Haikou, Hainan 571101 China

**Keywords:** Male infertility, Y chromosome microdeletion, AZF microdeletion, Reproductive hormones, Azoospermia, Oligozoospermia

## Abstract

**Background:**

Male infertility has become a global health problem, and genetic factors are one of the essential causes. Y chromosome microdeletion is the leading genetic factor cause of male infertility. The objective of this study is to investigate the correlation between male infertility and Y chromosome microdeletions in Hainan, the sole tropical island province of China.

**Methods:**

We analyzed the semen of 897 infertile men from Hainan in this study. Semen analysis was measured according to WHO criteria by professionals at the Department of Reproductive Medicine, the First Affiliated Hospital of Hainan Medical University, where samples were collected. Y chromosome AZF microdeletions were confirmed by detecting six STS markers using multiple polymerase chain reactions on peripheral blood DNA. The levels of reproductive hormones, including FSH, LH, PRL, T, and E_2_, were quantified using the enzyme-linked immunosorbent assay (ELISA).

**Results:**

The incidence of Y chromosome microdeletion in Hainan infertile men was 7.13%. The occurrence rate of Y chromosome microdeletion was 6.69% (34/508) in the oligozoospermia group and 7.71% (30/389) in the azoospermia group. The deletion of various types in the AZF subregion was observed in the group with azoospermia, whereas no AZFb deletion was detected in the oligozoospermia group. Among all patients with microdeletions, the deletion rate of the AZFc region was the higher at 68.75% (44 out of 64), followed by a deletion rate of 6.25% (4 out of 64) for the AZFa region and a deletion rate of 4.69% (3 out of 64) for the AZFb region. The deletion rate of the AZFa region was significantly higher in patients with azoospermia than in patients with oligozoospermia (0.51% vs. 0.39%, *p* < 0.001). In comparison, the deletion rate of the AZFc region was significantly higher in patients with oligozoospermia (3.08% vs. 6.30%, *p* < 0.001). Additionally, the AZFb + c subregion association deletion was observed in the highest proportion among all patients (0.89%, 8/897), followed by AZFa + b + c deletion (0.56%, 5/897), and exclusively occurred in patients with azoospermia. Hormone analysis revealed FSH (21.63 ± 2.01 U/L vs. 10.15 ± 0.96 U/L, *p* = 0.001), LH (8.96 ± 0.90 U/L vs. 4.58 ± 0.42 U/L, *p* < 0.001) and PRL (263.45 ± 21.84 mIU/L vs. 170.76 ± 17.10 mIU/L, *p* = 0.002) were significantly increased in azoospermia patients with microdeletions. Still, P and E_2_ levels were not significantly different between the two groups.

**Conclusions:**

The incidence of AZF microdeletion can reach 7.13% in infertile men in Hainan province, and the deletion of the AZFc subregion is the highest. Although the Y chromosome microdeletion rate is distinct in different regions or populations, the regions mentioned above of the Y chromosome may serve an indispensable role in regulating spermatogenesis. The analysis of Y chromosome microdeletion plays a crucial role in the clinical assessment and diagnosis of male infertility.

## Background

The current state of male reproductive health presents a significant global public health challenge, with available data indicating that approximately 50% of infertility cases worldwide can be attributed to male factors [1]. Factors associated with male infertility include abnormal hormone levels, erectile dysfunction, presence of anti-sperm antibodies, varicocele, exposure to chemicals or radiation, and other factors. Most cases are attributed to genetic factors [2, 3].

**Fig. 1:**
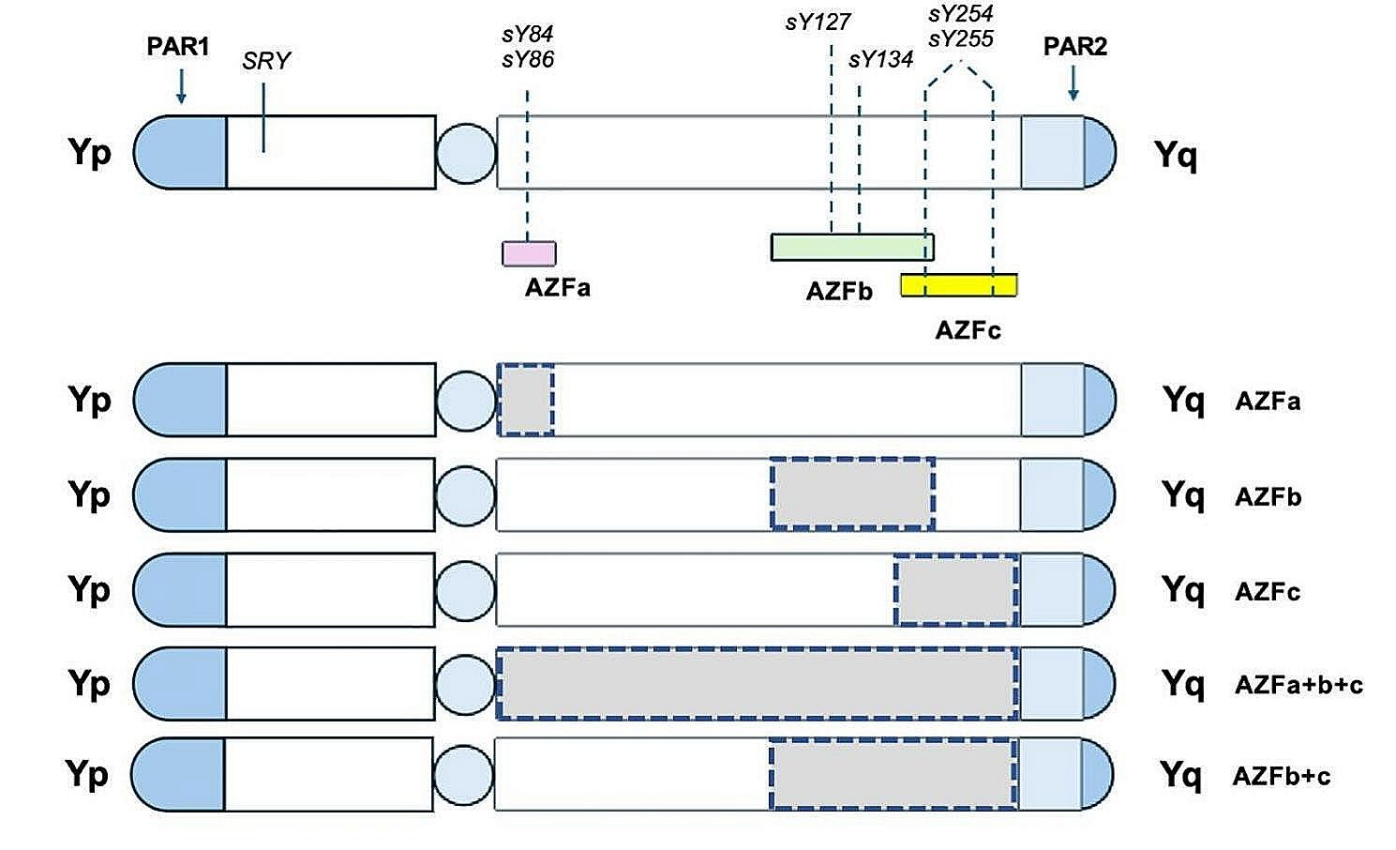
The Y-chromosome microdeletion model and the EAA/EMQN guidelines recommended sequence-tagged site (STS)

As early as 1976, *Tiepolo et al.* first found that azoospermia was associated with microdeletions on the long arm of the Y chromosome and hypothesized that it was primarily related to microdeletions in the azoospermia factor (AZF) region of the long arm of the Y chromosome, region 1, band 1 (Yq11) [4]. The human Y chromosome is essential in sex determination and spermatogenesis [5]. The AZF region is situated on the long arm of the Y chromosome. It is primarily subdivided into the AZFa, AZFb, and AZFc regions, encompassing genes crucial in spermatogenesis and testicular development [6], shown in Fig. 1. Microdeletions in the AZF region have been observed in most patients diagnosed with severe oligozoospermia and non-obstructive azoospermia (NOA) [7, 8]. It is regarded as one of the essential genetic factors for male infertility. The screening for Y chromosome microdeletion is now routinely employed in the evaluation of male infertility, offering prognostic value for patients with azoospermia and severe oligozoospermia.

The latest guidelines from The European Association of Urology (EAU) and the American Society for Reproductive Medicine (ASRM) state that the prevalence rates of Y-chromosome microdeletions in patients with severe oligozoospermia are 3–7% and 5%, respectively. They recommend screening for Y-chromosome microdeletions in men with sperm counts below 5 million/ml [9, 10]. Additionally, several studies have demonstrated that oligozoospermia and azoospermia are accompanied by alterations in levels of follicle-stimulating hormone (FSH), luteinizing hormone (LH), and testosterone (T) [11].

The province of Hainan is China’s only tropical island province, and it has a tropical monsoon marine climate. There are more than 30 ethnic groups in Hainan, including Han, Li, Miao, and Hui, among which ethnic minorities account for 18.02% of the province’s population. In this study, we evaluated the prevalence of various types of Y chromosome microdeletions in a cohort of infertile men from the southernmost region of China and analyzed their hormonal profile. Furthermore, we will conduct a comparative analysis between our findings and the documented regional outcomes in the Asian region, as well as the internationally published results.

## Methods

### Study participants

This study retrospectively enrolled 897 infertile males who attended the Reproductive Center of the First Affiliated Hospital of Hainan Medical University as study subjects. All patients were not using contraception for more than one year and had regular sexual life but were infertile, without other congenital diseases, and were diagnosed with non-obstructive azoospermia or oligozoospermia.

### Semen analysis

Patients were abstinent for 3–7 days, and semen was collected by masturbation method to observe the liquefaction time and to measure sperm concentration and viability, as well as sperm morphology analysis. All patients underwent at least two routine semen analyses. The results were analyzed and diagnosed according to the WHO laboratory manual for examining and processing human semen (5th edition).

### Y-Chromosome microdeletion screening

Each participant’s peripheral blood sample was collected, genomic DNA was extracted using the phenol-chloroform method, and DNA concentration and purity were checked. Subsequently, following the EAA/EMQN guidelines, screening was conducted using multiplex real-time PCR technology with six pairs of primers specific to sequence-tagged sites (STSs) in the AZF region: sY84 and sY86 in the AZFa region, sY127 and sY134 in the AZFb region, and sY254 and sY255 in the AZFc region. The sex-determining region of Y (SRY) was used as the internal control. PCR amplification products were qualified using agarose electrophoresis, and the results were observed and analyzed using an Alphalmager 2200 gel imaging system. Primers were used for SRY, as shown in Table 1.

**Table 1:** Sequence-tagged sites and primer sequences for Y chromosome microdeletion analysis

Region	STS	Probe	Sequence (5’ to 3’)	length (nt)
Yp	SRY	Forward	CCTCTTTGTTTTAAGGAAGAAAGGA	480
		Reverse	AATCACCTAGCAACTGATGCATTTA	
AZFa	sY84	Forward	AGAAGGGTCCTGAAAGCAGGT	324
		Reverse	GCCTACTACCTGGAGGCTTC	
	sY86	Forward	GTGACACACAGACTATGCTTC	318
		Reverse	ACACACAGAGGGACAACCCT	
AZFb	sY127	Forward	GGCTCACAAACGAAAAGAAA	274
		Reverse	CTGCAGGCAGTAATAAGGGA	
	sY134	Forward	GTCTGCCTCACCATAAAACG	301
		Reverse	ACCACTGCCAAAACTTTCAA	
AZFc	sY254	Forward	GGGTGTTACCAGAAGGCAAA	380
		Reverse	GAACCGTATCTACCAAAGCAGC	
	sY255	Forward	GTTACAGGATTCGGCGTGAT	126
		Reverse	CTCGTCATGTGCAGCCAC	

### Hormonal analysis

The peripheral blood sample was collected from each patient and centrifuged to collect their serum. ELISA assay was used to measure reproductive hormones: follicle-stimulating hormone (FSH), luteinizing hormone (LH), prolactin (PRL), progesterone (P), estrogen (E_2_), and testosterone (T).

### Assisted reproductive technology (ART)

Sperm were visible in the patient’s semen, and we selected the sperm among them. While the patient is azoospermia, Microdissection Testicular Sperm Extraction (mTESE) is performed to take sperm. If sperm is absent after mTESE, we recommend the patient accept the donated sperm. Controlled ovarian hyperstimulation was conducted using a gonadotropin-releasing hormone (GnRH) agonist based on a long protocol to obtain enough oocytes. Oocyte retrieval was performed 35–36 h after hCG injection, and all metaphase II oocytes underwent intracytoplasmic sperm injection (ICSI). Embryo transfer was performed according to the actual situation.

### Statistical analysis

Data were expressed as the mean ± the standard deviation (SD) or number (percentage %). Comparisons between outcome groups were performed using Student’s t-test for continuous variables and Chi-square test or Fisher’s exact test for categorical variables. Statistical significance was considered when *p* ≤ 0.05.

## Results

A total of 897 infertile men from Hainan Province, diagnosed with oligozoospermia or azoospermia and excluding chromosomal abnormalities, were included in the study. According to the semen analysis results, the patients were divided into the oligozoospermia group (508 cases) and the azoospermia group (389 cases). Y chromosome microdeletions were detected in 64 patients, with an overall incidence of 7.13% (64/897). The occurrence rate of Y chromosome microdeletion was 6.69% (34/508) in the oligozoospermia group and 7.71% (30/389) in the azoospermia group, respectively. As shown in Table 2, the deletion of various types in the AZF subregion was observed in the group with azoospermia, whereas no AZFb deletion was detected in the oligozoospermia group. Additionally, this study examined the prevalence of concurrent deletion of distinct subregions in all patients, with AZFb + c deletion exhibiting the highest frequency (0.89%, 8/897), followed by AZFa + b + c deletion (0.56%, 5/897). Multiple subregions were deleted in the azoospermia group, whereas no co-deletion was detected among patients with oligozoospermia. We observed a significantly higher deletion rate of the AZFa region in patients with azoospermia compared to those with oligozoospermia (0.51% vs. 0.39%, *p* < 0.001). Conversely, we found a significantly lower deletion rate of the AZFc region in patients with oligozoospermia (3.08% vs. 6.30%, *p* < 0.001, Table 2).

**Table 2:** The frequencies and types of Y chromosome microdeletions in infertile participants

Microdeletions	Azoospermia (*n* = 389)	Oligozoospermia (*n* = 508)	*p* value	Total *N*
microdeletions	7.71%(30/389)	6.69%(34/508)	*p* < 0.001	7.13%(64/897)
AZFa	0.51%(2/389)	0.39%(2/508)	*p* < 0.001	0.45%(4/897)
AZFb	0.77%(3/389)	0.00%(0/508)		0.33%(3/897)
AZFc	3.08%(12/389)	6.30%(32/508)	*p* < 0.001	4.91%(44/897)
AZFb + c	2.06%(8/389)	0.00%(0/508)		0.89%(8/897)
AZFa + b + c	1.29%(5/389)	0.00%(0/508)		0.56%(5/897)

**Fig. 2:**
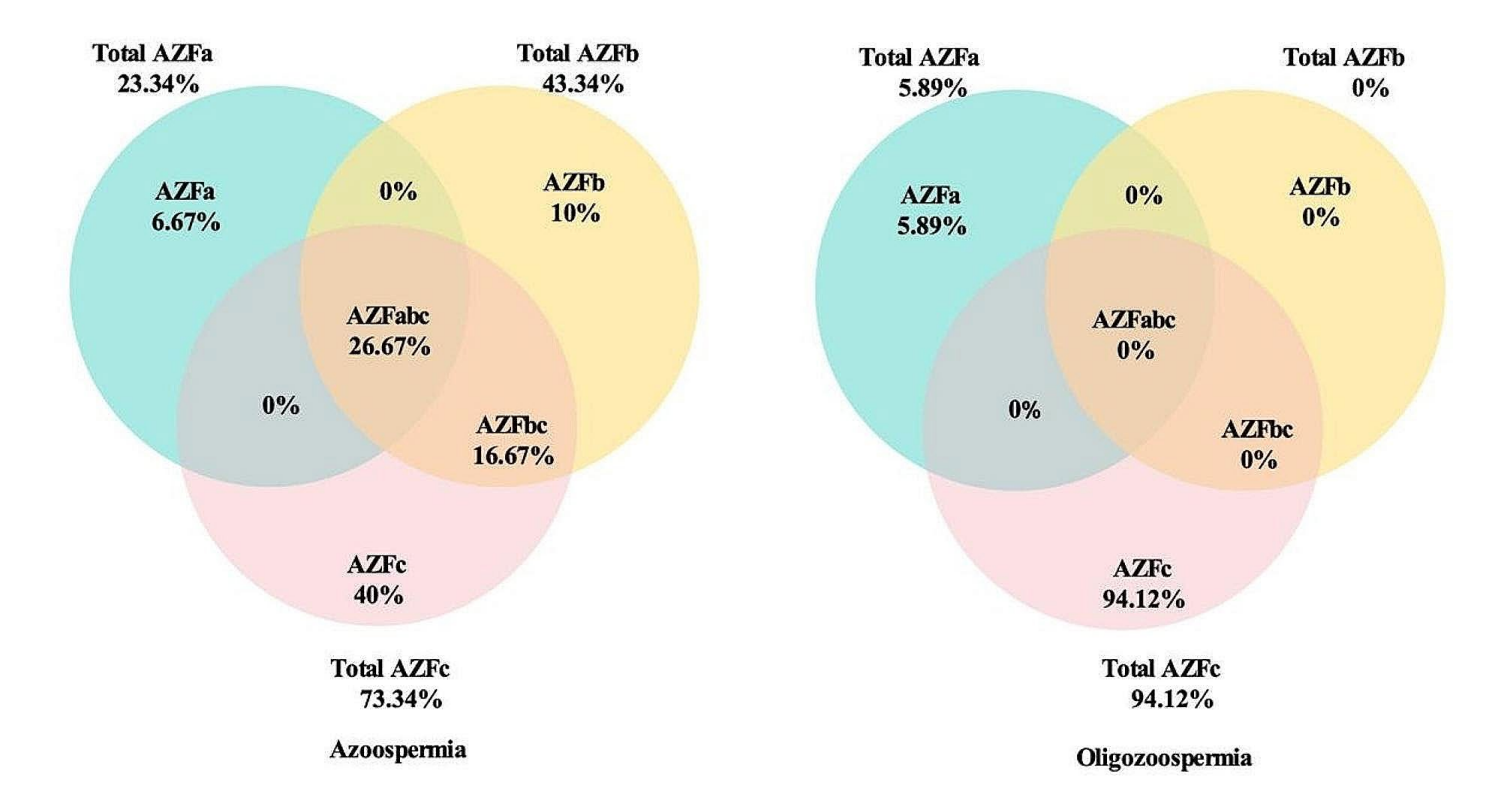
Diagram shows AZF microdeletion percentage in Azoospermia and Oligozoospermia patients. Total AZFa, AZFb, and AZFc show the percentage of deletion, including the combination

Among all patients with microdeletions, the deletion rate of AZFc region was the highest (68.75%, 44/64), followed by AZFa region deletion (6.25%, 4/64) and AZFb region deletion (4.69%, 3/64). The incidence of AZFc deletion was highest (83.33%, 25/30) among patients with azoospermia, including combined deletions (AZFb + c at 26.67% and AZFa + b + c at 16.67%). Total AZFb deletion had a higher rate than total AZFa region, while no cases of AZFa + b or AZFa + c deletions were detected. Similarly, among patients with oligozoospermia, the AZFc region exhibited the highest rate of total deletion (94.12%, *n* = 32). In contrast, no deletions were detected in the combined AZFb region and its various subregions, as illustrated in Fig. 2.

The serum levels of sex hormones (FSH, LH, PRL, T, E_2_, P) in the microdeletion patients were subsequently assessed. As shown in Table 3, compared to oligozoospermia patients with microdeletions, azoospermia patients with microdeletions exhibited significantly elevated levels of FSH (21.63 ± 2.01 U/L vs. 10.15 ± 0.96 U/L, *p* = 0.001), LH (8.96 ± 0.90 U/L vs. 4.58 ± 0.42 U/L, *p* < 0.001), and PRL (263.45 ± 21.84 mIU/L vs. 170.76 ± 17.10 mIU/L, *p* = 0.002). However, there were no significant differences in the levels of P and E_2_ between the two groups. Although T levels were higher in microdeletion oligospermia patients, the two groups had no statistical significance. 66.85% (44/64) of the patients with AZF deletions underwent Assisted Reproductive Technology (ART) procedures following diagnosis. 31.25% (20/64) of patients refused the donated sperm and opted not to proceed with ART.

**Table 3:** Comparison of the Mean hormonal levels in Azoospermic and Oligozoospermic infertile men with Y chromosome microdeletion

	Total	Azoospermia	Oligozoospermia	*p* value
Age (y)	33.15 ± 0.60	33.37 ± 1.01	32.97 ± 0.71	*p* = 0.021
FSH (U/L)	16.65 ± 1.46	21.63 ± 2.01	10.15 ± 0.96	*p* = 0.001
LH (U/L)	7.07 ± 0.65	8.96 ± 0.90	4.58 ± 0.42	*p* < 0.001
P (nmol/L)	1.91 ± 0.19	2.03 ± 0.13	1.78 ± 0.50	*p* = 0.638
PRL (mIU/L)	221.33 ± 16.14	263.45 ± 21.84	170.76 ± 17.10	*p* = 0.002
T (nmol/L)	23.18 ± 10.67	12.12 ± 0.91	37.13 ± 24.35	*p* = 0.060
E_2_(pmol/L)	106.38 ± 6.84	107.14 ± 7.17	101.60 ± 12.05	*p* = 0.605

## Discussion

Genetic factors are one of the critical factors leading to male infertility, including chromosome karyotype abnormality, gene copy number variation, single gene mutation, and Y chromosome microdeletion, among which the most common are karyotype abnormality and Y chromosome microdeletion [12]. The findings of several studies indicate that approximately 8–10% of infertile males in China exhibit Y chromosome microdeletions [13–16]. However, no studies with a substantial sample size have been published in the southernmost region of China.

In this study, we evaluated the prevalence of Y chromosome microdeletions in male infertility among the Hainan population, China’s sole tropical island province. A total of 897 infertile men from Hainan Province, diagnosed with oligozoospermia or azoospermia and excluding chromosomal abnormalities, were included in the study. Y chromosome microdeletions were detected in 64 patients, with an overall incidence of 7.13% (64/897). The rate of Y chromosome microdeletion in infertile males in Hainan was generally lower than in other regions of China [8] and the world, including Iraqi (47.8%) [17], Morocco (18.82%) [18], India (10.02%) [8] and Korea (10.93%) [19] but higher than in Turkey (6.82%) [20] (Table 4). It may be a unique group characteristic of the Hainan population in China. The deletion of the AZF region is typically regarded as Y chromosome microdeletion and is being gradually considered by specialists. The incidence of Y chromosome microdeletion in infertile men varies significantly among different laboratories due to the utilization of diverse population methodologies and STSs [21–23]. Therefore, it is imperative to integrate multiple influencing factors to comprehensively describe and elucidate the occurrence of Y chromosome microdeletions across diverse regions.

**Table 4:** Studies in Some of the Asia countries and regions with different frequencies

References	Year	Region	Study population	Frequency
*Wang et al.* [14]	2010	Northeastern China	305	9.2%
*Waseem et al.* [8]	2020	Indian	379	10.02%
*Liu et al.* [15]	2007	Guangzhou China	175	10.90%
*Jafari et al.* [45]	2022	Iranian	200	7.5%
*Sha et al.* [16]	2020	Eastern China	201	10.95%
*Al-Janabi et al.* [17]	2020	Iraqi	90	47.8%
*Birowo et al.* [46]	2017	Indonesian	71	15.49%
*Zhu et al.* [13]	2017	Southern China	1808	8.3%
This Study	2023	Hainan China	897	7.13%

The AZF region of the Y chromosome is further divided into three subregions: AZFa, AZFb, and AZFc. Regarding localization, the AZFa region is situated at the proximal end of the long arm of the Y chromosome, closest to the centromere. The AZFc region is located at the distal end, while the AZFb region lies between these two subregions (Fig. 1). Additionally, specific fragments within the AZFb region overlap with those in the AZFc region [24]. The deletion of distinct subregions results in varying sterility phenotypes. The present study investigated the effects of single and combined subregion deletion in infertile males. We found that the most common type of AZF microdeletion in infertile men in Hainan Province was AZFc deletion (4.91%), and there was a significant difference in incidence between oligozoospermia and azoospermia, i.e., infertile men with AZFc deletion were more frequently found to have oligozoospermia. The previous data indicated that the highest incidence of AZF microdeletion in infertile men was AZFc deletion [18, 20, 25–27]. However, some studies have reported different results. The recent study revealed a higher prevalence of AZFa subregion deletion in microdeletion infertile men, significantly correlated with the oligozoospermia phenotype [28]. Although much of the present data still suggests that AZFa deletions are not the most common type in male infertility patients compared with other kinds of AZF deletion [29, 30], it has been reported that AZFa deletion can lead to the most severe phenotype, which in the case of complete AZFa region deletion occurs as Sertoli Cell Only Syndrome (SCOS), in which there are no sperm cells at all in the testicular convoluted tubules, and only Sertoli cells are seen [31–33]. The reported prevalence of SCOS in azoospermia patients ranges from 26.3–57.8% [34, 35]. The etiologies of human SCOS are diverse, encompassing Y chromosome microdeletions, cryptorchidism, chromosomal disorders, cytotoxic drugs, radiation exposure, and viral infections [36].

In our study, we identified four infertility patients with AZFa deletions. Two patients exhibited complete AZFa deletions (i.e., deletions at both sY84 and sY86 STS sites) and consistently presented with azoospermia. The other two patients showed deletions only at the sY86 STS site, without the sY84 deletion, which was considered partial AZFa deletion and exhibited oligozoospermia. A recent study reported that approximately 61.3% of 75 males with severe oligozoospermia or azoospermia had AZFa region deletions, confirming a significant association between partial or complete AZFa deletions and male infertility and spermatogenic failure, with partial AZFa deletions occurring more frequently than complete deletions [37]. However, another study reported that microdeletions in the AZFa region are associated with diverse phenotypes, including azoospermia, oligozoospermia, and normozoospermia [38]. Patients with complete AZFa deletions and Sertoli cell-only syndrome (SCOS) cannot retrieve sperm via microdissection testicular sperm extraction (mTESE) for assisted reproductive techniques such as intracytoplasmic sperm injection (ICSI). Thus, mTESE is not recommended clinically [39]. Instead, donor sperm should be considered for assisted reproductive treatments of these patients. However, for patients with partial deletions who may present with oligozoospermia, mTESE followed by ICSI is a viable option. Still, the patient must be fully informed of the potential of inheriting the same deletion in the male of his offspring.

This study found two types of co-deletion of AZF subregions, AZFb + c and AZFa + b + c. The incidence of AZFb + c subregion combined deletion is higher in infertile men, followed by AZFa + b + c deletion. These two types of combined microdeletion were only detected in patients with azoospermia, not in oligozoospermia, consistent with previous reports [19]. It was shown that AZFb + c subregion deletion may be most closely related to the azoospermia phenotype. Azoospermia and oligozoospermia patients should perform Y chromosome deletion screening before testicular sperm extraction (TESE) or intracytoplasmic sperm injection (ICSI). If they are found in the region of AZFa, AZFb + c, or AZFa + b + c complete deletion, TESE should not be recommended. If these partial deletion patients have a strong desire for fertility, can we perform mTESE to win some opportunities for patients? This question is worth discussing and analyzing. Hainan Island has more than 30 ethnic groups, including Han, Li, Miao, and Hui, among which ethnic minorities account for 18.02% of the province’s population. Minority groups have more traditional ideas and a strong desire to have children. With fully informed consent, can we take some proactive ways to assist sperm retrieval in patients with AZF deletion? We need more scientific evidence and support.

The hypothalamic-pituitary-testicular axis mainly regulates testicular spermatogenesis and changes in reproductive hormones will affect male reproductive function [40]. This study further analyzed the sex hormone levels of infertile men with AZF microdeletion in Hainan. It aimed to explore the relationship between sex hormone levels and different sterility phenotypes caused by AZF microdeletion. We found a significant increase in serum FSH (21.63 ± 2.01 U/L vs. 10.15 ± 0.96 U/L) and LH (8.96 ± 0.90 U/L vs. 4.58 ± 0.42 U/L) levels in AZF microdeletion azoospermia patients compared to AZF microdeletion oligospermia patients. This finding is consistent with hormonal analysis results from the previous report [28]. Normal spermatogenesis depends on the synergistic regulation of multiple sex hormones. Elevated FSH levels suggest a decline in testicular function [41]. Previous studies have also shown that infertile patients with microdeletions have significantly higher FSH and LH levels than those without [19]. In addition, we found that compared with oligozoospermia patients, the serum PRL levels of AZF microdeletion in azoospermia men were also significantly higher. A previous study revealed that serum PRL levels were significantly elevated in patients with azoospermia compared to healthy men, while patients with oligozoospermia were in between [42]. In the male reproductive system, the prominent role of PRL is to regulate the FSH receptor on the supporting cells and the LH receptor on the testicular interstitial cells to affect the reproductive function of the testis [43]. At the same time, serum PRL can inhibit the release of GnRH and decrease the secretion of both FSH and LH [44]. FSH, LH, and PRL levels may have specific diagnostic and predictive values for infertile men with AZF microdeletion.

## Conclusion

The province of Hainan is the sole tropical island province in China. The incidence of AZF microdeletion can reach 7.13% in infertile men in Hainan province, and the deletion of the AZFc subregion is the highest. Although the Y chromosome microdeletion rate is distinct in different regions or populations, the regions mentioned above of the Y chromosome may serve an indispensable role in regulating spermatogenesis. The analysis of Y chromosome microdeletion plays a crucial role in the clinical assessment and diagnosis of male infertility. Further studies will help to understand better and diagnose male infertility and provide a theoretical basis for the diagnosis and treatment of male infertility.

## Data Availability

The datasets used and/or analysed during the current study are available from the corresponding author on reasonable request.
